# Electronic Properties of Graphene Nano-Parallelograms: A Thermally Assisted Occupation DFT Computational Study

**DOI:** 10.3390/molecules29020349

**Published:** 2024-01-10

**Authors:** Sonai Seenithurai, Jeng-Da Chai

**Affiliations:** 1 Department of Physics, National Taiwan University, Taipei 10617, Taiwan; seenithurai@gmail.com; 2 Center for Theoretical Physics and Center for Quantum Science and Engineering, National Taiwan University, Taipei 10617, Taiwan; 3 Physics Division, National Center for Theoretical Sciences, Taipei 10617, Taiwan

**Keywords:** TAO-DFT, electronic properties, graphene nano-parallelograms, multi-reference character

## Abstract

In this computational study, we investigate the electronic properties of zigzag graphene nano-parallelograms (GNPs), which are parallelogram-shaped graphene nanoribbons of various widths and lengths, using thermally assisted occupation density functional theory (TAO-DFT). Our calculations revealed a monotonic decrease in the singlet–triplet energy gap as the GNP length increased. The GNPs possessed singlet ground states for all the cases examined. With the increase of GNP length, the vertical ionization potential and fundamental gap decreased monotonically, while the vertical electron affinity increased monotonically. Some of the GNPs studied were found to possess fundamental gaps in the range of 1–3 eV, lying in the ideal region relevant to solar energy applications. Besides, as the GNP length increased, the symmetrized von Neumann entropy increased monotonically, denoting an increase in the degree of the multi-reference character associated with the ground state GNPs. The occupation numbers and real-space representation of active orbitals indicated that there was a transition from the nonradical nature of the shorter GNPs to the increasing polyradical nature of the longer GNPs. In addition, the edge/corner localization of the active orbitals was found for the wider and longer GNPs.

## 1. Introduction

Carbon nanostructures are ubiquitous. This versatility comes from the ability of carbon to form bonds through sp, sp^2^, and sp^3^ hybridization. The different types of hybridization can lead to various allotropes and complexes. Carbon is also available in nature as diamond and graphite. It forms all organic matter and is the basis of life. It is also an essential element in many drugs [1,2]. The nanoforms of allotropes were first introduced by the discovery of C_60_ molecules [3,4], followed by the observation of carbon nanotubes [5], then followed by the isolation and many groundbreaking experiments on graphene [6,7]. All these nanostructures have various special properties, which are interesting and can be advantageous for specific applications. The series of carbon nanoallotropes has created a fresh search for new 0D, 1D, and 2D nanomaterials [2]. Among carbon nanomaterials, graphene, which is a 2D nanomaterial, stands out due to its fascinating properties and vast potential applications [7,8].

As graphene lacks a band gap, its finite nanostructures, such as graphene nanoribbons (GNRs), are also of great interest because of a wide range of possibilities to tune their properties for specific applications. GNRs can be thought of as quasi-1D nanostructures carved from graphene. GNRs can exhibit intriguing electronic, optical, and magnetic characteristics [9,10,11,12,13,14,15,16], which can be useful for many applications [16,17,18,19]. In general, GNRs can be classified into two main categories: zigzag GNRs and armchair GNRs, based on their edge patterns [12,13,14]. Both zigzag and armchair GNRs of various widths have been successfully synthesized [12]. There are also other types of GNRs with different kinds of edge structures, such as the chevron, fjord, chiral, junction, cove, and gulf types of GNRs [20]. Although there is a rich literature on these electronic systems, ongoing theoretical and experimental studies aim to further explore the properties of GNRs.

**Figure 1:**
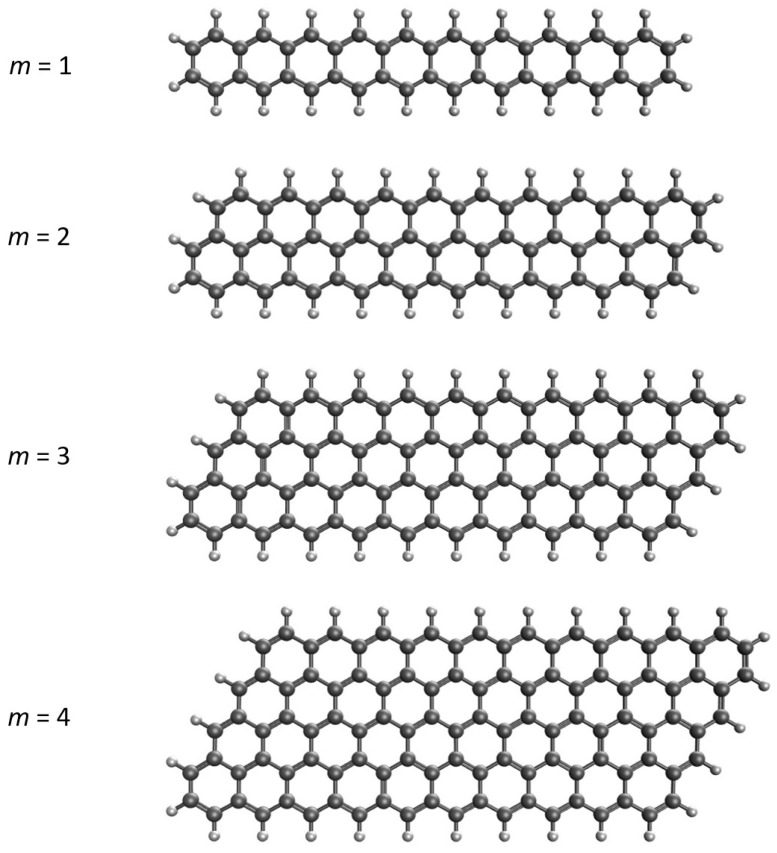
Structures of GNP[*m*,10] with *m* = 1 (i.e., 10-acene, containing 10 linearly fused benzene rings) and GNP[*m*,10] with *m* = 2–4 (containing *m* parallelly fused 10-acenes forming parallelograms).

Although zigzag and armchair GNRs have been extensively documented in the literature, reports on the parallelogram-shaped GNRs with zigzag edges (see Figure 1 for an illustration), denoted as graphene nano-parallelograms (GNPs), are relatively scarce. In this study, the narrowest GNPs, which are *n*-acenes (i.e., acenes with *n* linearly fused benzene rings), are labeled as GNP[1,*n*]; the second-narrowest GNPs are labeled as GNP[2,*n*], containing two parallelly fused *n*-acenes forming parallelograms; the third-narrowest GNPs are labeled as GNP[3,*n*], containing three parallelly fused *n*-acenes forming parallelograms, and so on. For GNP[*m*,*n*], the *m* value specifies the GNP width, and the *n* value specifies the GNP length.

On the experimental side, some GNPs, such as GNP[2,4], GNP[2,5], GNP[3,2], GNP[3,3], and GNP[3,4], were recently synthesized in molecular and crystalline forms [21,22]. Besides, for GNP[2,*n*] (with *n* = 3–5), their properties and lasing applications in the near-infrared (NIR) region have been reported [23]. In addition, Jousselin-Oba et al. [24] synthesized TIPS-peri-pentacenopentacene (TIPS-PPP) (a derivative of GNP[2,5]) and studied its optoelectronic properties. They showed that TIPS-PPP can be adopted as a highly efficient NIR emitter for NIR organic light-emitting-diode devices. Very recently, the derivatives of GNP[2,4] were found to possess interesting amplified spontaneous emission (ASE) properties, which can be useful for laser applications [25]. On the computational side, Omist et al. [26] reported the electronic properties of GNP[2,*n*] (with *n* = 3–7) and nitrogen-doped GNP[2,*n*], obtained with various electronic structure methods. Besides, Sandoval-Salinas et al. [27] performed electronic structure calculations to explore the electronic properties of GNP[*m*,*n*] (with m=n = 2–6). In addition, a recent computational study reported a few electronic properties of GNP[*m*,*n*] (with m=n = 2–4) [28].

However, several properties of GNP[*m*,*n*] with different widths and lengths remain unavailable on both the experimental and computational sides. On the experimental side, it remains challenging to realize the precise parallelogram-shaped graphene nanoribbons with smooth edges, especially for the larger GNPs (possibly due to the radical nature). On the computational side, GNPs, which have similar shapes as zigzag GNRs [29], can possess strong static correlation effects in their ground states. Accordingly, GNPs can be challenging systems for traditional electronic structure methods (as will be discussed later). Since investigating the finite-size effects of GNP[*m*,*n*] is essential to gain a comprehensive understanding of the width- and length-dependent electronic properties of GNP[*m*,*n*], this computational study aimed at exploring the electronic properties of GNP[*m*,*n*] with various values of *m* and *n*, using a non-traditional electronic structure method (i.e., suitable for studying the ground state properties of large electronic systems with strong static correlation effects).

Among existing electronic structure methods, Kohn–Sham density functional theory (KS-DFT) [30,31] is popular and valuable for investigating the ground state properties of electronic systems, especially for single-reference (SR) systems (i.e., electronic systems possessing an SR character in their ground states). In fact, KS-DFT has served as the backbone of theoretical condensed matter physics, quantum chemistry, and computational materials science [32]. For the sake of computational efficiency, KS-DFT calculations are commonly carried out using the local density approximation (LDA) [33,34] and generalized gradient approximation (GGA) [35] exchange-correlation (xc) energy functionals. However, KS-DFT with the LDA and GGA xc energy functionals can encounter severe problems for issues related to the self-interaction error, non-covalent interaction error, and static correlation error [36,37,38,39]. Among these qualitative errors, the self-interaction error can be reduced by the use of hybrid xc energy functionals [40,41,42], incorporating a fraction of Hartree–Fock exchange into the parent LDA or GGA xc energy functionals. Besides, a number of computationally efficient dispersion correction schemes [43,44], which can be directly added to the parent LDA or GGA xc energy functionals, are readily available for improving the description of non-covalent interactions. Nevertheless, the presence of strong static correlation effects in multi-reference (MR) systems (i.e., electronic systems possessing an MR character in their ground states) has posed a formidable challenge, particularly for KS-DFT with the conventional LDA, GGA, and hybrid xc energy functionals. Generally, SR electronic structure methods (e.g., KS-DFT with the conventional LDA, GGA, and hybrid xc energy functionals, as well as the Hartree–Fock theory) are unreliable for studying the ground state properties of MR systems.

Typically, to explore the ground state properties of MR systems, ab initio MR electronic structure methods [45,46,47,48,49,50,51], such as the MR configuration interaction (MRCI) methods and density-matrix renormalization group (DMRG) algorithms, are essential [52,53]. However, these methods can become impractical for large electronic systems due to the prohibitively high cost of performing ab initio MR electronic structure calculations. Accordingly, the demand for a reliable and efficient electronic structure method for large MR systems is very high.

Recently, thermally assisted occupation density functional theory (TAO-DFT) [54] has emerged as an effective solution for addressing the challenges posed by large MR systems. Generally, TAO-DFT focuses on improving the representability of the ground state electron density by incorporating fractional orbital occupation numbers, which can be efficiently computed using the Fermi–Dirac (FD) distribution function with some fictitious temperature θ . The LDA [54], GGA [55], global hybrid [56], and range-separated [56,57] exchange-correlation- θ (xc θ ) energy functionals (i.e., the combined xc and θ -dependent energy functionals [58]) can be incorporated in TAO-DFT. Besides, simple models for defining the optimal system-independent [59] and system-dependent [60] fictitious temperatures of an energy functional in TAO-DFT have been recently proposed. Note also that the difference among KS-DFT [30,31], TAO-DFT [54], and finite-temperature density functional theory (FT-DFT) [31,61] (i.e., three generally different electronic structure methods) has been properly discussed in a recent work [58].

Very recently, various TAO-DFT-related extensions, such as TAO-DFT-based ab initio molecular dynamics (TAO-AIMD) [62], TAO-DFT with the polarizable continuum model (TAO-PCM) [63], and a real-time extension of TAO-DFT (RT-TAO-DFT) [58], have been developed, expanding the capabilities of TAO-DFT to handle a more-diverse range of applications. Moreover, TAO-DFT has also been adopted to investigate the various properties (e.g., electronic properties [29,64,65,66,67,68], hydrogen storage properties [65], spectroscopic properties [62,69,70], and equilibrium thermodynamic properties [62]) of MR systems at the nanoscale.

Consequently, in this computational study, we utilized TAO-DFT to obtain the electronic properties of GNP[*m*,*n*] (with *m* = 1–4 and *n* = 2–30), including the singlet–triplet energy gaps, vertical electron affinities/ionization potentials, fundamental gaps, symmetrized von Neumann entropy, active orbital occupation numbers, and real-space representation of active orbitals.

## 2. Results and Discussion

### 2.1. Singlet–Triplet Energy Gap

For a neutral molecule, the singlet–triplet energy gap (ST gap) provides an understanding of the ground state nature and by how much the lowest singlet and triplet states are separated in the energy landscape. The ST gap can also be useful for understanding the radical nature of molecules [71,72,73,74,75], as well as for providing valuable information for photovoltaic applications [76].

Here, the ST gap ( $E_{\mathrm{ST}}$ ) of GNP[*m*,*n*] was calculated as the energy difference between the lowest singlet and triplet states of GNP[*m*,*n*] (evaluated on the respective optimized geometries):

(1) $$E_{\mathrm{ST}}=E_{\mathrm{UT}}-E_{\mathrm{US}},$$

where $E_{\mathrm{US}}$ / $E_{\mathrm{UT}}$ is the energy of the lowest singlet/triplet state of GNP[*m*,*n*], obtained with spin-unrestricted TAO-LDA.

**Figure 2:**
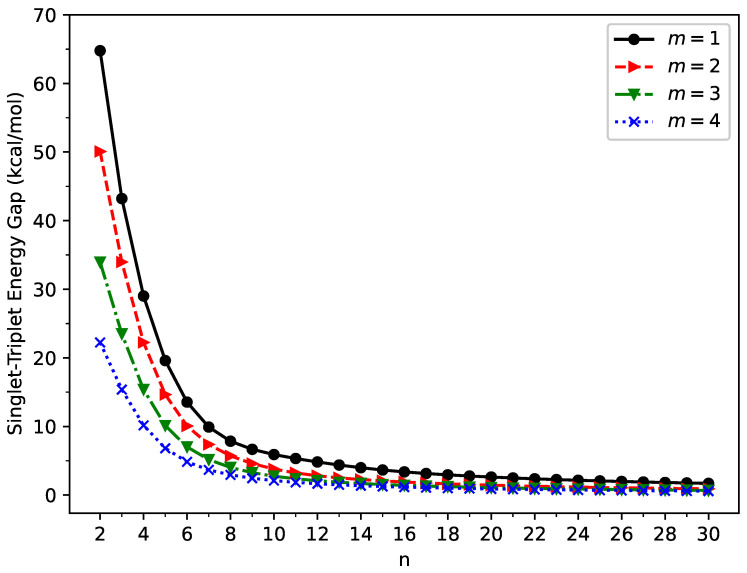
Singlet–triplet energy gap of GNP[*m*,*n*], calculated using spin-unrestricted TAO-LDA.

As presented in Figure 2, GNP[*m*,*n*] possess singlet ground states (also see Table S1 in the Supplementary Information (SI)) for all the cases examined (i.e., *m* = 1–4 and *n* = 2–30). The ST gap decreases monotonically as the GNP length increases. Since MR systems typically possess very small ST gaps, the longer GNPs can have the MR character in their ground states.

It is well known that, for the lowest singlet state of an MR system, the spin-symmetry constraint, which must be satisfied by an exact theory, can be violated by SR electronic structure methods (e.g., KS-DFT with the conventional LDA, GGA, and hybrid xc energy functionals, as well as the Hartree–Fock theory) [29,37,54,55,56,58,62,63,77]. In other words, spin-unrestricted and spin-restricted SR electronic structure calculations can yield different energies for the lowest singlet state of an MR system, leading to unphysical spin-symmetry-breaking effects. Here, to examine if this spin-symmetry constraint can be satisfied by TAO-LDA, spin-restricted TAO-LDA calculations were additionally carried out for the lowest singlet energies of GNP[*m*,*n*] (evaluated on the respective optimized geometries). Within the numerical accuracy considered in the present study, spin-unrestricted and spin-restricted TAO-LDA calculations essentially yield the same energies for the lowest singlet states of GNP[*m*,*n*] (with *m* = 1–4 and *n* = 2–30), indicating that essentially no unphysical spin-symmetry-breaking effects occur in our spin-unrestricted TAO-LDA solutions for all the cases examined.

### 2.2. Vertical Ionization Potential, Vertical Electron Affinity, and Fundamental Gap

The vertical ionization potential, vertical electron affinity, and fundamental gap of a neutral molecule in the ground state are important electronic properties. The vertical ionization potential is the energy change when an electron is removed from the neutral molecule (without changing its geometry), and the vertical electron affinity is the energy change when an electron is added to the neutral molecule (without changing its geometry). The fundamental gap is the difference between the vertical ionization potential and vertical electron affinity.

According to their definitions, on the spin-unrestricted TAO-LDA-optimized geometry of ground state GNP[*m*,*n*], we computed the vertical ionization potential:

(2) $${\mathrm{IP}}_{v}=E_{N-1}-E_{N},$$

vertical electron affinity:

(3) $${\mathrm{EA}}_{v}=E_{N}-E_{N+1},$$

and fundamental gap:

(4) $$E_{g}={\mathrm{IP}}_{v}-{\mathrm{EA}}_{v}$$

of ground state GNP[*m*,*n*], using multiple energy-difference calculations, with $E_{N}$ being the total energy of the *N*-electron molecule (i.e., GNP[*m*,*n*]) obtained with spin-unrestricted TAO-LDA.

**Figure 3:**
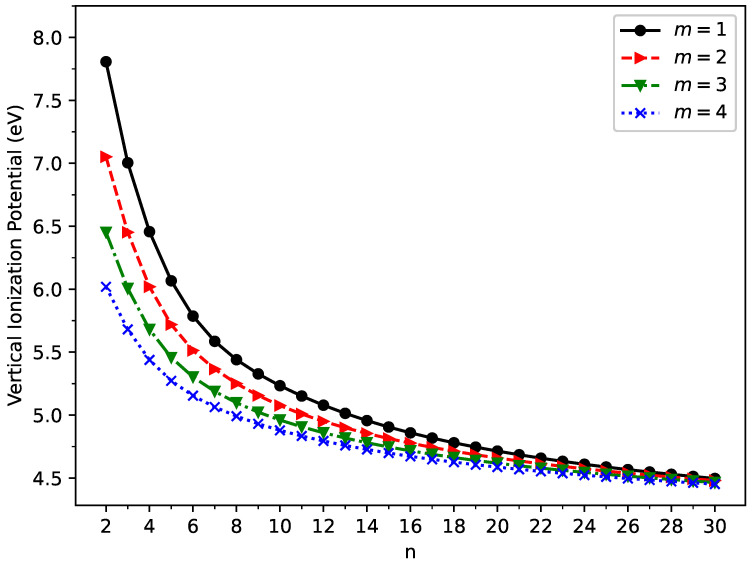
Vertical ionization potential for the ground state of GNP[*m*,*n*], calculated using spin-unrestricted TAO-LDA.

**Figure 4:**
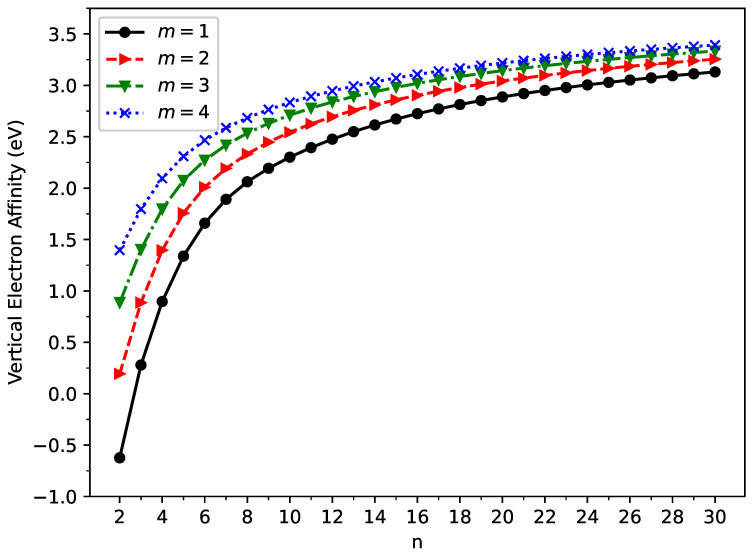
Vertical electron affinity for the ground state of GNP[*m*,*n*], calculated using spin-unrestricted TAO-LDA.

**Figure 5:**
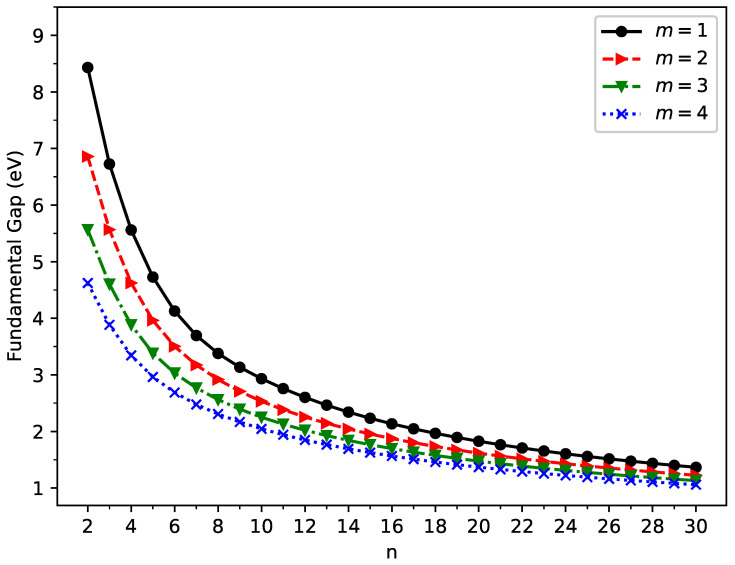
Fundamental gap for the ground state of GNP[*m*,*n*], calculated using spin-unrestricted TAO-LDA.

The vertical ionization potential ( ${\mathrm{IP}}_{v}$ ), vertical electron affinity ( ${\mathrm{EA}}_{v}$ ), and fundamental gap ( $E_{g}$ ) of ground state GNP[*m*,*n*] are shown in Figure 3, Figure 4, and Figure 5, respectively (also, see Tables S2–S5 in the SI). With increasing GNP length, the ${\mathrm{IP}}_{v}$ decreases monotonically and the ${\mathrm{EA}}_{v}$ increases monotonically, leading to a monotonically decreasing $E_{g}$ . These quantities are essential in materials science, spectroscopy, catalyst selection, etc., and are also important for the selection of candidate materials for electronic devices, solar cells, etc. Generally, electronic systems with the fundamental gaps in the range of 1–3 eV are desirable for photovoltaic applications. According to our TAO-LDA results, for all the cases studied in this work, GNP[1,*n*] (*n* = 10–30), GNP[2,*n*] (*n* = 8–30), GNP[3,*n*] (*n* = 7–30), and GNP[4,*n*] (*n* = 5–30) can be suitable candidates for photovoltaic applications.

### 2.3. Symmetrized von Neumann Entropy

To estimate the degree of the MR character associated with ground state GNP[*m*,*n*], we computed the symmetrized von Neumann entropy [55,56,77]:

(5) $$S_{\mathrm{vN}}=-\frac{1}{2}\sum_{\sigma =\alpha ,\beta }\sum_{i=1}^{\infty }\left\{f_{i,\sigma }\;\ln\left(f_{i,\sigma }\right)+\left(1-f_{i,\sigma }\right)\;\ln\left(1-f_{i,\sigma }\right)\right\},$$

where $f_{i,\sigma }$ (a number between 0 and 1) is the occupation number of the *i*th σ -spin (i.e., α -spin or β -spin) orbital, calculated by spin-unrestricted TAO-LDA [54], approximately yielding the *i*th σ -spin natural orbital occupation number [64,78]. As can be seen in Equation (5), a term with $f_{i,\sigma }$ = 0 or 1 has no contribution to $S_{\mathrm{vN}}$ , while a term with $f_{i,\sigma }$ significantly differing from 0 and 1 can cause a large increase in the $S_{\mathrm{vN}}$ . As a result, for an SR system, all $f_{i,\sigma }$ values should be very close to 0 or 1, and hence, $S_{\mathrm{vN}}$ should be vanishingly small. By contrast, for an MR system, some $f_{i,\sigma }$ values can significantly differ from 0 and 1, and hence, $S_{\mathrm{vN}}$ can become very large. While there are other quantitative measures [79,80] of the MR character of an electron system, the symmetrized von Neumann entropy ( $S_{\mathrm{vN}}$ ), which is closely related to the entropy contribution in TAO-DFT [54,55,56], can be readily obtained at no extra computational cost.

**Figure 6:**
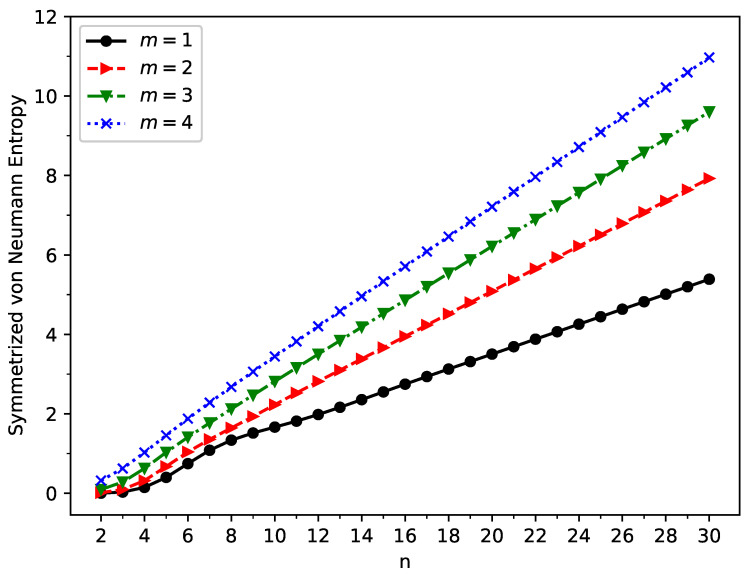
Symmetrized von Neumann entropy for the ground state of GNP[*m*,*n*], calculated using spin-unrestricted TAO-LDA.

As shown in Figure 6, the symmetrized von Neumann entropy ( $S_{\mathrm{vN}}$ ) of ground state GNP[*m*,*n*] increases monotonically with increasing GNP length (also, see Tables S2–S5 in the SI). This indicates that the degree of the MR character associated with ground state GNP[*m*,*n*] should generally increase as the GNP length increases.

### 2.4. Active Orbital Occupation Numbers

With increasing GNP length, to justify the increasing trend of $S_{\mathrm{vN}}$ of ground state GNP[*m*,*n*], in spin-unrestricted TAO-DFT, there should be more active spin-orbitals (i.e., spin-orbitals with considerable fractional occupation numbers (e.g., in the range of 0.1–0.9)) and/or the occupation numbers of active spin-orbitals are closer to 0.5. In spin-restricted TAO-DFT, this indicates that there should be more active orbitals (i.e., orbitals with occupation numbers in the range of 0.2–1.8) and/or the occupation numbers of active orbitals are closer to 1.

**Figure 7:**
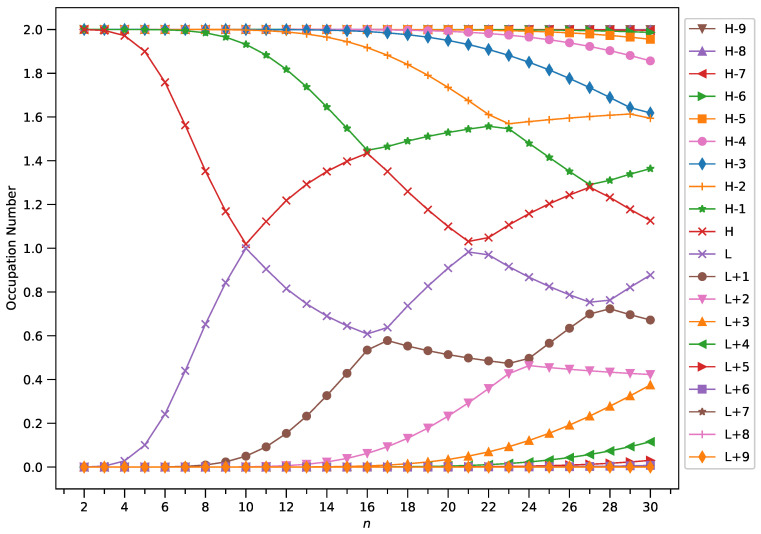
Occupation numbers of active orbitals (HOMO−9, HOMO−8, …, HOMO, LUMO, …, LUMO+8, and LUMO+9) for the ground state of GNP[1,*n*], calculated using spin-restricted TAO-LDA.

**Figure 8:**
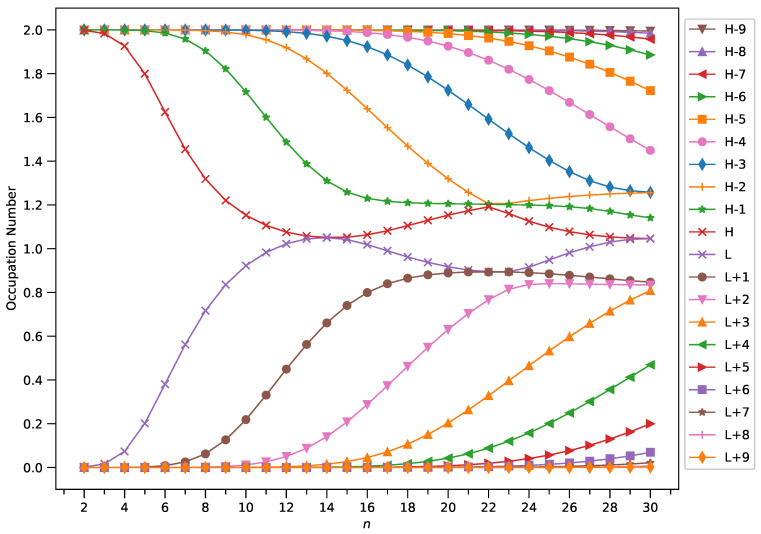
Occupation numbers of active orbitals (HOMO−9, HOMO−8, …, HOMO, LUMO, …, LUMO+8, and LUMO+9) for the ground state of GNP[2,*n*], calculated using spin-restricted TAO-LDA.

**Figure 9:**
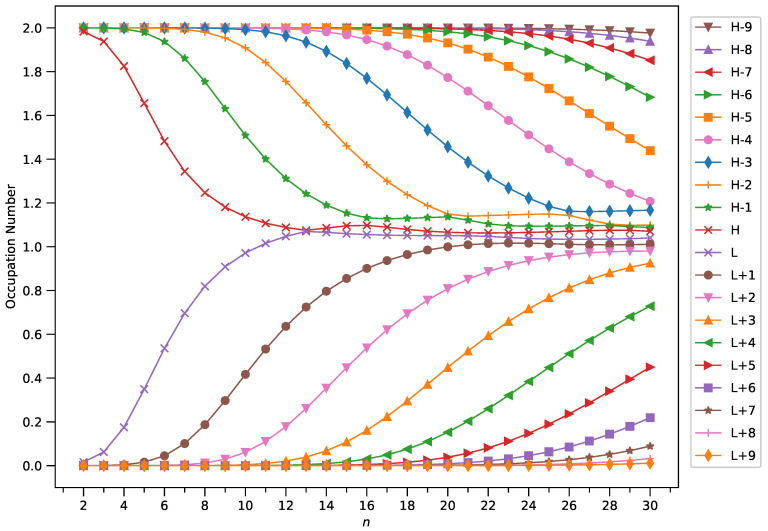
Occupation numbers of active orbitals (HOMO−9, HOMO−8, …, HOMO, LUMO, …, LUMO+8, and LUMO+9) for the ground state of GNP[3,*n*], calculated using spin-restricted TAO-LDA.

**Figure 10:**
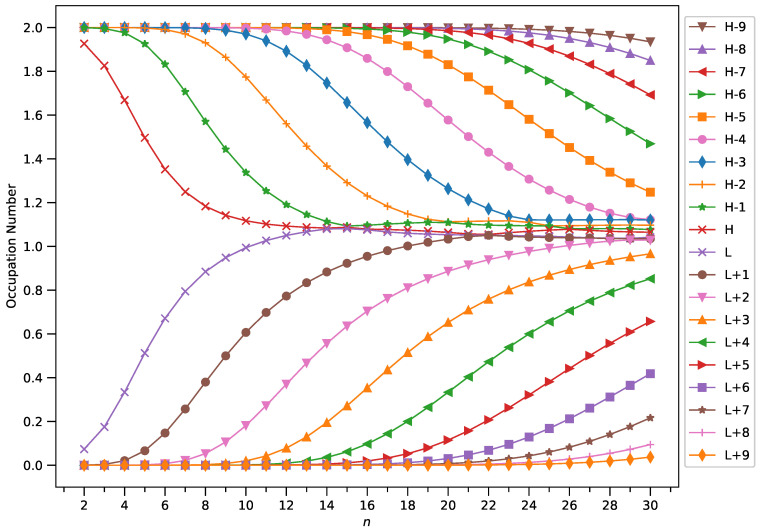
Occupation numbers of active orbitals (HOMO−9, HOMO−8, …, HOMO, LUMO, …, LUMO+8, and LUMO+9) for the ground state of GNP[4,*n*], calculated using spin-restricted TAO-LDA.

To examine this, we plotted the occupation numbers of active orbitals for the ground state of GNP[*m*,*n*], calculated using spin-restricted TAO-LDA (see Figure 7 (for *m* = 1), Figure 8 (for *m* = 2), Figure 9 (for *m* = 3), and Figure 10 (for *m* = 4)). For the ground state of GNP[*m*,*n*] (containing *N* electrons), the highest occupied molecular orbital (HOMO) is the (N/2) th orbital, the lowest unoccupied molecular orbital (LUMO) is the (N/2+1) th orbital, and so on. In the present study, for brevity, HOMO and LUMO are denoted as H and L, respectively.

A common observation is that the shorter GNPs possess a nonradical nature (i.e., all the orbital occupation numbers are very close to 0 or 2) and the longer GNPs possess an increasing polyradical nature (i.e., as the GNP length increases, there are more active orbitals and/or the occupation numbers of active orbitals are closer to 1). Therefore, as the GNP length increases, the general feature of a transition from the nonradical nature of the shorter GNPs to the increasing polyradical nature of the longer GNPs is observed for each GNP width examined (i.e., *m* = 1–4). For the wider GNPs, the evolution of the polyradical nature is faster.

### 2.5. Real-Space Representation of Active Orbitals

**Figure 11:**
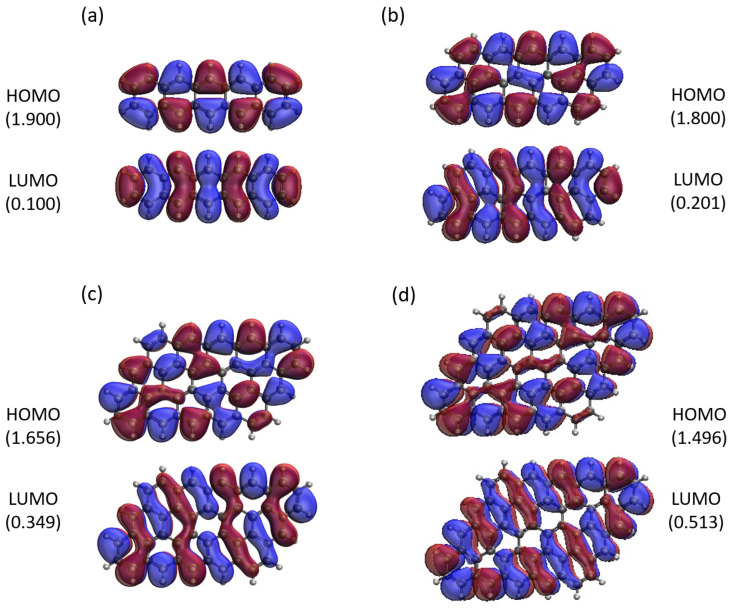
Real-space representation of active orbitals (HOMO and LUMO) for the ground state of GNP[*m*,5] with (**a**) m=1 , (**b**) m=2 , (**c**) m=3 , and (**d**) m=4 , at an isovalue of 0.02 e/Å^3^, calculated using spin-restricted TAO-LDA, where the orbital occupation numbers are shown in parentheses.

**Figure 12:**
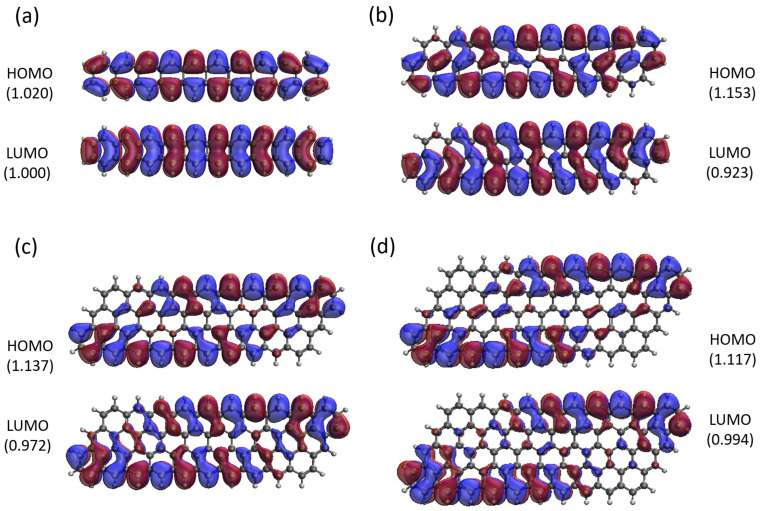
Real-space representation of active orbitals (HOMO and LUMO) for the ground state of GNP[*m*,10] with (**a**) m=1 , (**b**) m=2 , (**c**) m=3 , and (**d**) m=4 , at an isovalue of 0.02 e/Å^3^, calculated using spin-restricted TAO-LDA, where the orbital occupation numbers are shown in parentheses.

**Figure 13:**
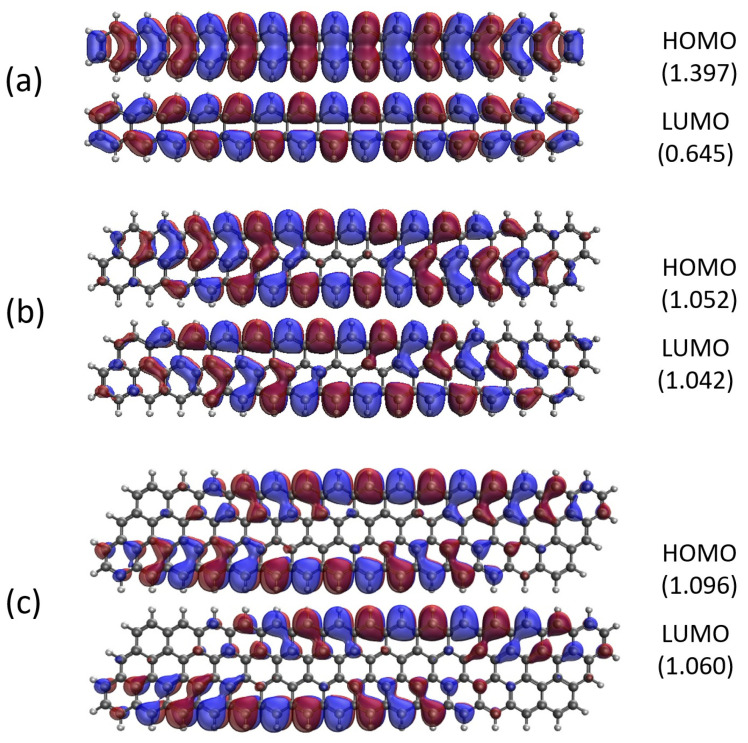

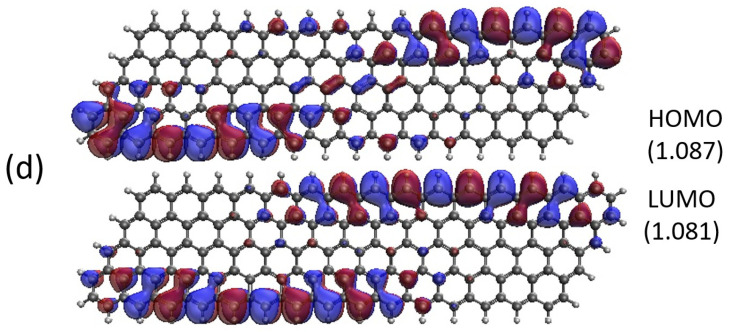
Real-space representation of active orbitals (HOMO and LUMO) for the ground state of GNP[*m*,15] with (**a**) m=1 , (**b**) m=2 , (**c**) m=3 , and (**d**) m=4 , at an isovalue of 0.02 e/Å^3^, calculated using spin-restricted TAO-LDA, where the orbital occupation numbers are shown in parentheses.

**Figure 14:**
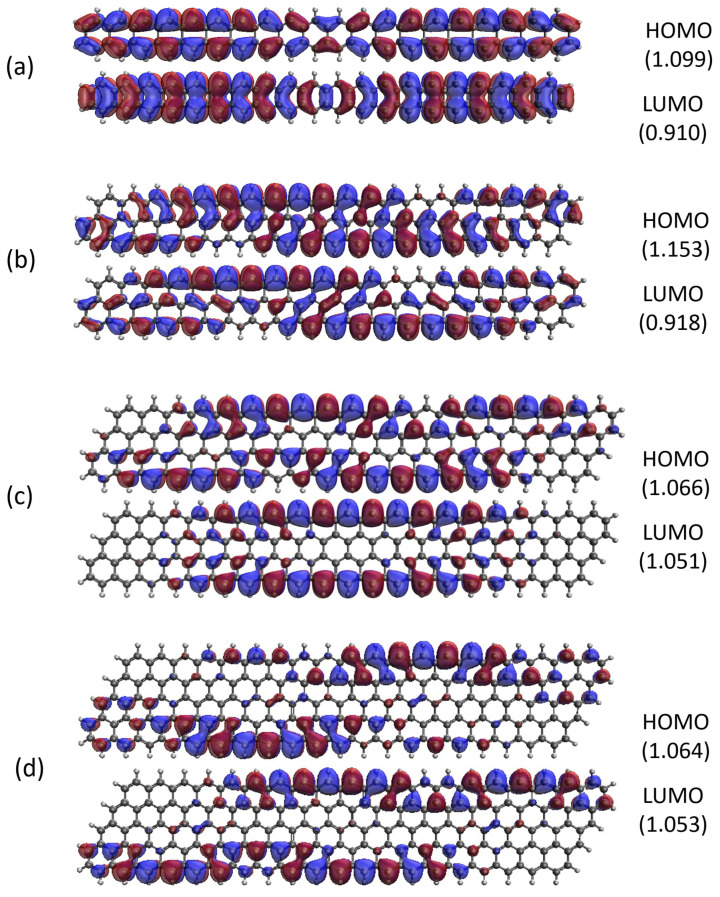
Real-space representation of active orbitals (HOMO and LUMO) for the ground state of GNP[*m*,20] with (**a**) m=1 , (**b**) m=2 , (**c**) m=3 , and (**d**) m=4 , at an isovalue of 0.02 e/Å^3^, calculated using spin-restricted TAO-LDA, where the orbital occupation numbers are shown in parentheses.

Here, we report the real-space representation of active orbitals (HOMO and LUMO) for the ground state of some representative GNP[*m*,*n*], including GNP[*m*,5] with *m* = 1–4 (see Figure 11), GNP[*m*,10] with *m* = 1–4 (see Figure 12), GNP[*m*,15] with *m* = 1–4 (see Figure 13), and GNP[*m*,20] with *m* = 1–4 (see Figure 14), obtained with spin-restricted TAO-LDA.

As shown, for a very narrow and short GNP (e.g., GNP[1,*n*] with *n* = 2–5), the HOMO and LUMO are delocalized over the entire GNP. However, as the width and length of the GNP (i.e., *m* and *n*) increase, the HOMO and LUMO of ground state GNP[*m*,*n*] have an increasing tendency to localize at the longer edges, as well as the corners of smaller interior angles. In contrast to the edge localization of active orbitals found for the wider and longer zigzag GNRs [29,77], the edge/corner localization of active orbitals for the wider and longer GNPs was observed in this study, possibly due to the parallelogram shapes of GNPs.

## 3. Materials and Methods

We performed all calculations with Q-Chem 4.4 [81], using the 6-31G(d) basis set. The electronic properties of GNP[*m*,*n*] (with *m* = 1–4 and *n* = 2–30) were computed using TAO-LDA (i.e., TAO-DFT with the LDA xc θ energy functional) with the recommended fictitious temperature θ = 7 mEh [54].

## 4. Conclusions

In conclusion, we report the electronic properties of GNP[*m*,*n*] (with *m* = 1–4 and *n* = 2–30) obtained with TAO-DFT. As the longer GNPs exhibit a significant MR character (e.g., polyradical nature), KS-DFT with the conventional LDA, GGA, and hybrid xc energy functionals can lead to unreliable results. Besides, because of the large electronic systems considered, performing ab initio MR electronic structure calculations can be impractical due to the prohibitively high cost. Owing to its nice compromise between efficiency and accuracy, TAO-DFT appears to be an ideal electronic structure method for this computational study.

Here, we summarize our key findings in a concise manner. First, according to TAO-DFT, the ST gap decreased monotonically with increasing GNP length, and the GNPs possessed singlet ground states for all the cases examined. Second, as the GNP length increased, the vertical ionization potential and fundamental gap decreased monotonically, whereas the vertical electron affinity and symmetrized von Neumann entropy increased monotonically. Third, similar to the findings of previous studies on zigzag GNRs [29,77], the shorter GNPs possessed a nonradical nature, and the longer GNPs possessed an increasing polyradical nature.

As a result, there was a transition from the nonradical nature of the shorter GNPs to the increasing polyradical nature of the longer GNPs. Besides, the evolution of polyradical nature was faster for the wider GNPs. However, in contrast to the edge localization of active orbitals found for the wider and longer zigzag GNRs [29,77], we observed the edge/corner localization of active orbitals for the wider and longer GNPs in this study, possibly due to the parallelogram shapes of the GNPs.

## Data Availability

The numerical data supporting the findings of this study are available from the authors upon appropriate request.

## Supplementary Materials

The following Supporting Information can be downloaded at: www.mdpi.com/xxx/s1.

## References

[1] Hirsch A. (2010). The era of carbon allotropes. Nat. Mater..

[2] Georgakilas V., Perman J.A., Tucek J., Zboril R. (2015). Broad family of carbon nanoallotropes: Classification, chemistry, and applications of fullerenes, carbon dots, nanotubes, graphene, nanodiamonds, and combined superstructures. Chem. Rev..

[3] Kroto H.W., Heath J.R., O’Brien S.C., Curl R.F., Smalley R.E. (1985). C60: Buckminsterfullerene. Nature.

[4] Meirzadeh E., Evans A.M., Rezaee M., Milich M., Dionne C.J., Darlington T.P., Bao S.T., Bartholomew A.K., Handa T., Rizzo D.J. (2023). A few-layer covalent network of fullerenes. Nature.

[5] Iijima S. (1991). Helical microtubules of graphitic carbon. Nature.

[6] Novoselov K.S., Geim A.K., Morozov S.V., Jiang D.E., Zhang Y., Dubonos S.V., Grigorieva I.V., Firsov A.A. (2004). Electric field effect in atomically thin carbon films. Science.

[7] Geim A.K., Novoselov K.S. (2007). The rise of graphene. Nat. Mater..

[8] Madurani K.A., Suprapto S., Machrita N.I., Bahar S.L., Illiya W., Kurniawan F. (2020). Progress in graphene synthesis and its application: History, challenge and the future outlook for research and industry. ECS J. Solid State Sci. Technol..

[9] Houtsma R.K., de la Rie J., Stöhr M. (2021). Atomically precise graphene nanoribbons: Interplay of structural and electronic properties. Chem. Soc. Rev..

[10] Gu Y., Qiu Z., Müllen K. (2022). Nanographenes and graphene nanoribbons as multitalents of present and future materials science. J. Am. Chem. Soc..

[11] Han M.Y., Özyilmaz B., Zhang Y., Kim P. (2007). Energy band-gap engineering of graphene nanoribbons. Phys. Rev. Lett..

[12] Friedrich N., Menchón R.E., Pozo I., Hieulle J., Vegliante A., Li J., Sánchez-Portal D., Penã D., Garcia-Lekue A., Pascual J.I. (2022). Addressing electron spins embedded in metallic graphene nanoribbons. ACS Nano.

[13] Son Y.W., Cohen M.L., Louie S.G. (2006). Energy gaps in graphene nanoribbons. Phys. Rev. Lett..

[14] Kimouche A., Ervasti M.M., Drost R., Halonen S., Harju A., Joensuu P.M., Sainio J., Liljeroth P. (2015). Ultra-narrow metallic armchair graphene nanoribbons. Nat. Commun..

[15] Jiang S., Neuman T., Boeglin A., Scheurer F., Schull G. (2023). Topologically localized excitons in single graphene nanoribbons. Science.

[16] Kumar S., Pratap S., Kumar V., Mishra R.K., Gwag J.S., Chakraborty B. (2023). Electronic, transport, magnetic, and optical properties of graphene nanoribbons and their optical sensing applications: A comprehensive review. Luminescence.

[17] Wang H., Wang H.S., Ma C., Chen L., Jiang C., Chen C., Xie X., Li A.P., Wang X. (2021). Graphene nanoribbons for quantum electronics. Nat. Rev. Phys..

[18] Saraswat V., Jacobberger R.M., Arnold M.S. (2021). Materials science challenges to graphene nanoribbon electronics. ACS Nano.

[19] Luo H., Yu G. (2022). Preparation, bandgap engineering, and performance control of graphene nanoribbons. Chem. Mater..

[20] Yano Y., Mitoma N., Ito H., Itami K. (2019). A quest for structurally uniform graphene nanoribbons: Synthesis, properties, and applications. J. Org. Chem..

[21] Gu Y., Wu X., Gopalakrishna T.Y., Phan H., Wu J. (2018). Graphene-like molecules with four zigzag edges. Ang. Chem. Int. Ed..

[22] Gu Y., Tullimilli Y.G., Feng J., Phan H., Zeng W., Wu J. (2019). peri-Acenoacenes. Chem. Comm..

[23] Muñoz–Mármol R., Gordillo F., Bonal V., Villalvilla J.M., Boj P.G., Quintana J.A., Ross A.M., Paternò G.M., Scotognella F., Lanzani G. (2021). Near-Infrared lasing in four-zigzag edged nanographenes by 1D versus 2D electronic *π*-conjugation. Adv. Funct. Mater..

[24] Jousselin-Oba T., Mamada M., Wright K., Marrot J., Adachi C., Yassar A., Frigoli M. (2022). Synthesis, aromaticity, and application of peri-pentacenopentacene: Localized representation of benzenoid aromatic compounds. Angew. Chem. Int. Ed..

[25] Gu Y., Muñoz-Mármol R., Fan W., Han Y., Wu S., Li Z., Bonal V., Villalvilla J.M., Quintana J.A., Boj P.G. (2022). Peri-acenoacene for solution processed distributed feedback laser: The effect of 1,2-oxaborine doping. Adv. Opt. Mater..

[26] Omist A., Ricci G., Derradji A., Pérez-Jiménez A.J., San-Fabián E., Olivier Y., Sancho-Garcia J.C. (2021). Peri-acenoacene molecules: Tuning of the singlet and triplet excitation energies by modifying their radical character. Phys. Chem. Chem. Phys..

[27] Sandoval-Salinas M.E., Bernabeu-Cabañero R., Pérez-Jiménez A.J., San-Fabián E., Sancho-García J.C. (2023). Electronic structure of rhombus-shaped nanographenes: System size evolution from closed- to open-shell ground states. Phys. Chem. Chem. Phys..

[28] Hauwali N.U.J., Syuhada I., Rosikhin A., Winata T. (2021). Fundamental properties of parallelogram graphene nanoflakes: A first principle study. Mat. Today Proc..

[29] Wu C.-S., Chai J.-D. (2015). Electronic properties of zigzag graphene nanoribbons studied by TAO-DFT. J. Chem. Theory Comput..

[30] Hohenberg P., Kohn W. (1964). Inhomogeneous electron gas. Phys. Rev..

[31] Kohn W., Sham L.J. (1965). Self-consistent equations including exchange and correlation effects. Phys. Rev..

[32] Teale A.M., Helgaker T., Savin A., Adamo C., Aradi B., Arbuznikov A.V., Ayers P.W., Baerends E.J., Barone V., Calaminici P. (2022). DFT Exchange: Sharing perspectives on the workhorse of quantum chemistry and materials science. Phys. Chem. Chem. Phys..

[33] Dirac P.A.M. (1930). Note on exchange phenomena in the Thomas atom. Proc. Camb. Philos. Soc..

[34] Perdew J.P., Wang Y. (1992). Accurate and simple analytic representation of the electron-gas correlation energy. Phys. Rev. B.

[35] Perdew J.P., Burke K., Ernzerhof M. (1996). Generalized gradient approximation made simple. Phys. Rev. Lett..

[36] Kümmel S., Kronik L. (2008). Orbital-dependent density functionals: Theory and applications. Rev. Mod. Phys..

[37] Cohen A.J., Mori-Sánchez P., Yang W. (2008). Insights into current limitations of density functional theory. Science.

[38] Engel E., Dreizler R.M. (2011). Density Functional Theory: An Advanced Course.

[39] Cohen A.J., Mori-Sánchez P., Yang W. (2012). Challenges for density functional theory. Chem. Rev..

[40] Becke A.D. (1993). A new mixing of Hartree–Fock and local density-functional theories. J. Chem. Phys..

[41] Becke A.D. (1993). Density-functional thermochemistry. III. The role of exact exchange. J. Chem. Phys..

[42] Stephens P.J., Devlin F.J., Chabalowski C.F., Frisch M.J. (1994). Ab initio calculation of vibrational absorption and circular dichroism spectra using density functional force fields. J. Phys. Chem..

[43] Grimme S. (2006). Semiempirical GGA-type density functional constructed with a long-range dispersion correction. J. Comput. Chem..

[44] Grimme S., Hansen A., Brandenburg J.G., Bannwarth C. (2016). Dispersion-corrected mean-field electronic structure methods. Chem. Rev..

[45] Andersson K., Malmqvist P.-Å., Roos B.O. (1992). Second-order perturbation theory with a complete active space self-consistent field reference function. J. Chem. Phys..

[46] Hachmann J., Dorando J.J., Aviles M., Chan G.K.L. (2007). The radical character of the acenes: A density matrix renormalization group study. J. Chem. Phys..

[47] Gidofalvi G., Mazziotti D.A. (2008). Active-space two-electron reduced-density-matrix method: Complete active-space calculations without diagonalization of the *N*-electron hamiltonian. J. Chem. Phys..

[48] Mizukami W., Kurashige Y., Yanai T. (2013). More *π* electrons make a difference: Emergence of many radicals on graphene nanoribbons studied by ab initio DMRG theory. J. Chem. Theory Comput..

[49] Gryn’ova G., Coote M.L., Corminboeuf C. (2015). Theory and practice of uncommon molecular electronic configurations. WIREs Comput. Mol. Sci..

[50] Fosso-Tande J., Nguyen T.-S., Gidofalvi G., DePrince III A.E. (2016). Large-scale variational two-electron reduced-density-matrix-driven complete active space self-consistent field methods. J. Chem. Theory Comput..

[51] Piris M. (2017). Global method for electron correlation. Phys. Rev. Lett..

[52] Goli V.D.P., Prodhan S., Mazumdar S., Ramasesha S. (2016). Correlated electronic properties of some graphene nanoribbons: A DMRG study. Phys. Rev. B.

[53] Hagymási I., Legeza Ö. (2016). Entanglement, excitations, and correlation effects in narrow zigzag graphene nanoribbons. Phys. Rev. B.

[54] Chai J.-D. (2012). Density functional theory with fractional orbital occupations. J. Chem. Phys..

[55] Chai J.-D. (2014). Thermally-assisted-occupation density functional theory with generalized-gradient approximations. J. Chem. Phys..

[56] Chai J.-D. (2017). Role of exact exchange in thermally-assisted-occupation density functional theory: A proposal of new hybrid schemes. J. Chem. Phys..

[57] Xuan F., Chai J.-D., Su H. (2019). Local density approximation for the short-range exchange free energy functional. ACS Omega.

[58] Tsai H.-Y., Chai J.-D. (2023). Real-time extension of TAO-DFT. Molecules.

[59] Chen B.-J., Chai J.-D. (2022). TAO-DFT fictitious temperature made simple. RSC Adv..

[60] Lin C.-Y., Hui K., Chung J.-H., Chai J.-D. (2017). Self-consistent determination of the fictitious temperature in thermally-assisted-occupation density functional theory. RSC Adv..

[61] Mermin N.D. (1965). Thermal properties of the inhomogeneous electron gas. Phys. Rev..

[62] Li S., Chai J.-D. (2020). TAO-DFT-based ab initio molecular dynamics. Front. Chem..

[63] Seenithurai S., Chai J.-D. (2023). TAO-DFT with the polarizable continuum model. Nanomaterials.

[64] Yeh C.-N., Chai J.-D. (2016). Role of Kekulé and non-Kekulé structures in the radical character of alternant polycyclic aromatic hydrocarbons: A TAO-DFT study. Sci. Rep..

[65] Seenithurai S., Chai J.-D. (2016). Effect of Li adsorption on the electronic and hydrogen storage properties of acenes: A dispersion-corrected TAO-DFT study. Sci. Rep..

[66] Tönshoff C., Bettinger H.F. (2021). Pushing the limits of acene chemistry: The recent surge of large acenes. Chem. Eur. J..

[67] Gupta D., Omont A., Bettinger H.F. (2021). Energetics of formation of cyclacenes from 2,3-didehydroacenes and implications for astrochemistry. Chem. Eur. J..

[68] Nieman R., Carvalho J.R., Jayee B., Hansen A., Aquino A.J., Kertesz M., Lischka H. (2023). Polyradical character assessment using multireference calculations and comparison with density-functional derived fractional occupation number weighted density analysis. Phys. Chem. Chem. Phys..

[69] Hanson-Heine M.W.D. (2020). Static correlation in vibrational frequencies studied using thermally-assisted-occupation density functional theory. Chem. Phys. Lett..

[70] Hanson-Heine M.W.D. (2022). Static electron correlation in anharmonic molecular vibrations: A hybrid TAO-DFT study. J. Phys. Chem. A.

[71] Su Y., Wang X., Wang L., Zhang Z., Wang X., Song Y., Power P.P. (2016). Thermally controlling the singlet–triplet energy gap of a diradical in the solid state. Chem. Sci..

[72] Yu L., Wu Z., Xie G., Zhong C., Zhu Z., Cong H., Ma D., Yang C. (2016). Achieving a balance between small singlet–triplet energy splitting and high fluorescence radiative rate in a quinoxaline-based orange-red thermally activated delayed fluorescence emitter. Chem. Commun..

[73] Smith M.B., Michl J. (2010). Singlet fission. Chem. Rev..

[74] Zhou J., Liu Q., Feng W., Sun Y., Li F. (2015). Upconversion luminescent materials: Advances and applications. Chem. Rev..

[75] Romero N.A., Nicewicz D.A. (2016). Organic photoredox catalysis. Chem. Rev..

[76] Xia J., Sanders S.N., Cheng W., Low J.Z., Liu J., Campos L.M., Sun T. (2017). Singlet fission: Progress and prospects in solar cells. Adv. Mater..

[77] Rivero P., Jiménez-Hoyos C.A., Scuseria G.E. (2013). Entanglement and polyradical nature of polycyclic aromatic hydrocarbons predicted by projected Hartree–Fock theory. J. Phys. Chem. B.

[78] Löwdin P.-O., Shull H. (1956). Natural orbitals in the quantum theory of two-electron systems. Phys. Rev..

[79] Takatsuka K., Fueno T., Yamaguchi K. (1978). Distribution of odd electrons in ground state molecules. Theor. Chim. Acta.

[80] Head-Gordon M. (2003). Characterizing unpaired electrons from the one-particle density matrix. Chem. Phys. Lett..

[81] Shao Y., Gan Z., Epifanovsky E., Gilbert A.T., Wormit M., Kussmann J., Lange A.W., Behn A., Deng J., Feng X. (2015). Advances in molecular quantum chemistry contained in the Q-Chem 4 program package. Mol. Phys..

