# Supplementary material for: Electronic Properties of Graphene Nano-Parallelograms: A Thermally Assisted Occupation DFT Computational Study
Source: Molecules. 2024 Jan 10;29(2):0. doi: 10.3390/molecules29020349 (PMC11154290; doi:10.3390/molecules29020349)
Supplement: Supplementary file 1 [file molecules-29-00349-s001.zip › molecules-2804734-supplementary.pdf]

LIST OF TABLES

S1 Singlet-triplet energy gap E_{ST} (in kcal/mol) of GNP[m,n] (m = 1–4), calculated using spin-unrestricted TAO-LDA. 2

S2 Vertical ionization potential IP_v (in eV), vertical electron affinity EA_v (in eV), fundamental gap E_g (in eV), and symmetrized von Neumann entropy S_{vN} for the ground state of GNP[1,n], calculated using spin-unrestricted TAO-LDA. . 3

S3 Vertical ionization potential IP_v (in eV), vertical electron affinity EA_v (in eV), fundamental gap E_g (in eV), and symmetrized von Neumann entropy S_{vN} for the ground state of GNP[2,n], calculated using spin-unrestricted TAO-LDA. . 4

S4 Vertical ionization potential IP_v (in eV), vertical electron affinity EA_v (in eV), fundamental gap E_g (in eV), and symmetrized von Neumann entropy S_{vN} for the ground state of GNP[3,n], calculated using spin-unrestricted TAO-LDA. . 5

S5 Vertical ionization potential IP_v (in eV), vertical electron affinity EA_v (in eV), fundamental gap E_g (in eV), and symmetrized von Neumann entropy S_{vN} for the ground state of GNP[4,n], calculated using spin-unrestricted TAO-LDA. . 6

TABLES

TABLE S1. Singlet-triplet energy gap E_{ST} (in kcal/mol) of GNP[m,n] (m = 1–4), calculated using spin-unrestricted TAO-LDA.

n	E_{ST} (GNP[1,n])	E_{ST} (GNP[2,n])	E_{ST} (GNP[3,n])	E_{ST} (GNP[4,n])
2	64.77	50.08	33.96	22.24
3	43.21	33.97	23.50	15.38
4	29.01	22.25	15.38	10.16
5	19.60	14.64	10.13	6.82
6	13.55	10.07	7.01	4.84
7	9.91	7.38	5.17	3.67
8	7.84	5.71	4.02	2.94
9	6.66	4.59	3.26	2.45
10	5.90	3.79	2.75	2.11
11	5.32	3.22	2.37	1.85
12	4.82	2.81	2.09	1.65
13	4.38	2.50	1.86	1.49
14	3.98	2.26	1.69	1.35
15	3.65	2.06	1.54	1.25
16	3.37	1.90	1.42	1.15
17	3.14	1.76	1.31	1.07
18	2.94	1.64	1.22	1.00
19	2.77	1.53	1.14	0.94
20	2.62	1.44	1.08	0.89
21	2.49	1.36	1.02	0.84
22	2.37	1.28	0.96	0.80
23	2.25	1.22	0.91	0.76
24	2.15	1.16	0.87	0.73
25	2.06	1.11	0.83	0.69

26	1.98	1.06	0.79	0.66
27	1.90	1.01	0.76	0.64
28	1.83	0.97	0.73	0.61
29	1.76	0.93	0.70	0.59
30	1.70	0.90	0.68	0.57

TABLE S2. Vertical ionization potential IP_v (in eV), vertical electron affinity EA_v (in eV), fundamental gap E_g (in eV), and symmetrized von Neumann entropy S_{vN} for the ground state of GNP[1,n], calculated using spin-unrestricted TAO-LDA.

n	IP_v	EA_v	E_g	S_{vN}
2	7.81	-0.62	8.43	0.00
3	7.00	0.28	6.73	0.03
4	6.46	0.90	5.56	0.15
5	6.07	1.34	4.73	0.40
6	5.79	1.66	4.13	0.75
7	5.59	1.89	3.69	1.08
8	5.44	2.06	3.38	1.34
9	5.33	2.19	3.13	1.52
10	5.23	2.30	2.93	1.67
11	5.15	2.39	2.76	1.82
12	5.08	2.48	2.60	1.98
13	5.01	2.55	2.46	2.16
14	4.96	2.61	2.34	2.36
15	4.91	2.67	2.23	2.55
16	4.86	2.72	2.13	2.75
17	4.82	2.77	2.05	2.94
18	4.78	2.81	1.97	3.13
19	4.75	2.85	1.89	3.31
20	4.71	2.89	1.83	3.50
21	4.68	2.92	1.76	3.69

22	4.66	2.95	1.71	3.88
23	4.63	2.98	1.65	4.07
24	4.61	3.00	1.60	4.26
25	4.59	3.03	1.56	4.44
26	4.57	3.05	1.52	4.63
27	4.55	3.07	1.47	4.82
28	4.53	3.09	1.44	5.01
29	4.51	3.11	1.40	5.20
30	4.50	3.13	1.37	5.39

TABLE S3. Vertical ionization potential IP_v (in eV), vertical electron affinity EA_v (in eV), fundamental gap E_g (in eV), and symmetrized von Neumann entropy S_{vN} for the ground state of GNP[2,n], calculated using spin-unrestricted TAO-LDA.

n	IP_v	EA_v	E_g	S_{vN}
2	7.05	0.19	6.86	0.02
3	6.45	0.89	5.57	0.09
4	6.02	1.40	4.62	0.32
5	5.72	1.76	3.96	0.67
6	5.51	2.01	3.50	1.04
7	5.37	2.19	3.17	1.36
8	5.25	2.33	2.92	1.64
9	5.16	2.45	2.71	1.93
10	5.08	2.54	2.53	2.23
11	5.01	2.62	2.38	2.53
12	4.95	2.69	2.26	2.82
13	4.90	2.76	2.14	3.10
14	4.85	2.81	2.04	3.38
15	4.81	2.86	1.95	3.67
16	4.78	2.90	1.87	3.95
17	4.74	2.94	1.80	4.23

18	4.71	2.98	1.73	4.52
19	4.69	3.01	1.67	4.80
20	4.66	3.04	1.62	5.09
21	4.64	3.07	1.56	5.37
22	4.61	3.10	1.52	5.65
23	4.59	3.12	1.47	5.94
24	4.57	3.14	1.43	6.22
25	4.56	3.17	1.39	6.51
26	4.54	3.19	1.35	6.79
27	4.52	3.20	1.32	7.07
28	4.51	3.22	1.29	7.36
29	4.49	3.24	1.25	7.64
30	4.48	3.25	1.23	7.93

TABLE S4. Vertical ionization potential IP_v (in eV), vertical electron affinity EA_v (in eV), fundamental gap E_g (in eV), and symmetrized von Neumann entropy S_{vN} for the ground state of GNP[3,n], calculated using spin-unrestricted TAO-LDA.

n	IP_v	EA_v	E_g	S_{vN}
2	6.45	0.89	5.57	0.09
3	6.01	1.40	4.60	0.28
4	5.68	1.80	3.88	0.62
5	5.46	2.07	3.38	1.03
6	5.30	2.27	3.03	1.41
7	5.19	2.42	2.77	1.77
8	5.10	2.54	2.56	2.12
9	5.02	2.63	2.39	2.47
10	4.96	2.71	2.25	2.82
11	4.91	2.78	2.13	3.16
12	4.86	2.84	2.02	3.50
13	4.82	2.89	1.93	3.84

14	4.78	2.94	1.84	4.18
15	4.75	2.98	1.77	4.52
16	4.72	3.02	1.70	4.86
17	4.69	3.05	1.63	5.20
18	4.66	3.09	1.58	5.54
19	4.64	3.12	1.52	5.88
20	4.62	3.14	1.48	6.22
21	4.60	3.17	1.43	6.55
22	4.58	3.19	1.39	6.89
23	4.56	3.21	1.35	7.23
24	4.54	3.23	1.31	7.57
25	4.53	3.25	1.28	7.91
26	4.51	3.27	1.24	8.25
27	4.50	3.29	1.21	8.58
28	4.49	3.30	1.18	8.92
29	4.48	3.32	1.16	9.26
30	4.46	3.33	1.13	9.60

TABLE S5. Vertical ionization potential IP_v (in eV), vertical electron affinity EA_v (in eV), fundamental gap E_g (in eV), and symmetrized von Neumann entropy S_{vN} for the ground state of GNP[4,n], calculated using spin-unrestricted TAO-LDA.

n	IP_v	EA_v	E_g	S_{vN}
2	6.02	1.40	4.62	0.32
3	5.68	1.80	3.88	0.62
4	5.44	2.10	3.34	1.03
5	5.27	2.31	2.96	1.46
6	5.15	2.47	2.69	1.88
7	5.06	2.59	2.48	2.28
8	4.99	2.68	2.31	2.68
9	4.93	2.76	2.17	3.06

10	4.88	2.83	2.05	3.44
11	4.83	2.89	1.94	3.82
12	4.79	2.95	1.85	4.20
13	4.76	2.99	1.77	4.58
14	4.73	3.03	1.69	4.96
15	4.70	3.07	1.63	5.33
16	4.67	3.11	1.57	5.71
17	4.65	3.14	1.51	6.09
18	4.63	3.17	1.46	6.46
19	4.61	3.19	1.41	6.84
20	4.59	3.22	1.37	7.21
21	4.57	3.24	1.33	7.59
22	4.55	3.26	1.29	7.96
23	4.54	3.28	1.26	8.34
24	4.52	3.30	1.22	8.72
25	4.51	3.32	1.19	9.09
26	4.50	3.33	1.16	9.47
27	4.48	3.35	1.13	9.84
28	4.47	3.36	1.11	10.22
29	4.46	3.38	1.08	10.59
30	4.45	3.39	1.06	10.97
